# Cucumber mosaic virus and its 2b protein alter emission of host volatile organic compounds but not aphid vector settling in tobacco

**DOI:** 10.1186/s12985-017-0754-0

**Published:** 2017-05-03

**Authors:** Trisna Tungadi, Simon C. Groen, Alex M. Murphy, Adrienne E. Pate, Javaid Iqbal, Toby J. A. Bruce, Nik J. Cunniffe, John P. Carr

**Affiliations:** 1 Department of Plant Sciences, University of Cambridge, Downing Street, Cambridge, CB2 3EA UK; 2 Rothamsted Research, Harpenden, Hertfordshire AL5 2JQ UK; 3 Present Address: Department of Biology, Center for Genomics and Systems Biology, New York University, New York, NY 10003 USA

**Keywords:** RNA silencing, 2b protein, Systemic induced resistance, Induced susceptibility, Plant-herbivore interactions, Plant-virus interactions, Secondary metabolism, Semiochemical, Jasmonates, *Cucumovirus*, *Potyvirus*

## Abstract

**Background:**

Aphids, including the generalist herbivore *Myzus persicae*, transmit cucumber mosaic virus (CMV). CMV (strain Fny) infection affects *M. persicae* feeding behavior and performance on tobacco (*Nicotiana tabacum*), *Arabidopsis thaliana* and cucurbits in varying ways. In Arabidopsis and cucurbits, CMV decreases host quality and inhibits prolonged feeding by aphids, which may enhance virus transmission rates. CMV-infected cucurbits also emit deceptive, aphid-attracting volatiles, which may favor virus acquisition. In contrast, aphids on CMV-infected tobacco (cv. Xanthi) exhibit increased survival and reproduction. This may not increase transmission but might increase virus and vector persistence within plant communities. The CMV 2b counter-defense protein diminishes resistance to aphid infestation in CMV-infected tobacco plants. We hypothesised that in tobacco CMV and its 2b protein might also alter the emission of volatile organic compounds that would influence aphid behavior.

**Results:**

Analysis of headspace volatiles emitted from tobacco plants showed that CMV infection both increased the total quantity and altered the blend produced. Furthermore, experiments with a CMV *2b* gene deletion mutant (CMV∆2b) showed that the 2b counter-defense protein influences volatile emission. Free choice bioassays were conducted where wingless *M. persicae* could choose to settle on infected or mock-inoculated plants under a normal day/night regime or in continual darkness. Settling was recorded at 15 min, 1 h and 24 h post-release. Statistical analysis indicated that aphids showed no marked preference to settle on mock-inoculated versus infected plants, except for a marginally greater settlement of aphids on mock-inoculated over CMV-infected plants under normal illumination.

**Conclusions:**

CMV infection of tobacco plants induced quantitative and qualitative changes in host volatile emission and these changes depended in part on the activity of the 2b counter-defense protein. However, CMV-induced alterations in tobacco plant volatile emission did not have marked effects on the settling of aphids on infected versus mock-inoculated plants even though CMV-infected plants are higher quality hosts for *M. persicae*.

**Electronic supplementary material:**

The online version of this article (doi:10.1186/s12985-017-0754-0) contains supplementary material, which is available to authorized users.

## Background

*Cucumber mosaic virus* (CMV) is the type species of the genus *Cucumovirus* and has an extremely broad host range comprising in excess of 1200 plant species [1, 2]. CMV is vectored by over 70 aphid species. Among the best-studied CMV vectors is the generalist herbivore *Myzus persicae* (common names: peach-potato or green peach aphid) [1, 2]. *M. persicae* attacks a diverse range of plants and is found in most parts of the world [3]. CMV transmission by aphids is non-persistent. This means that virus particles are retained for relatively short time periods (minutes to a few hours) in the mouthparts (stylet) of the aphid, are acquired from infected plants within a few seconds of feeding, and are released rapidly during salivation [1, 2, 4]. The loose binding of CMV particles to the stylet is mediated by specific amino acid residues on the viral coat protein and unknown receptor(s) within the common duct of the stylet [5, 6]. Several recent studies also suggest that viral gene products other than coat proteins and helper factors also influence virus transmission but through indirect means (reviewed in refs. [7–9]).

A CMV gene product that plays an indirect role in virus transmission is the 2b counter-defense protein [10]. On tobacco (*Nicotiana tabacum* cv. Xanthi) plants infected with the Fny strain of CMV, the reproduction and survival of *M. persicae* is enhanced [10, 11]. In contrast, it was found that on tobacco plants infected with a *2b* gene deletion mutant of the Fny strain of CMV (CMV∆2b) the aphid death rate was increased and reproduction of the insects was diminished [10]. Thus, in the tobacco cultivar Xanthi the effects of CMV infection, or the activity of one or more CMV gene products, have the potential to elicit antibiosis against aphids; as seen when plants are infected with CMV∆2b. Ziebell and colleagues [10] postulated that the 2b protein inhibited this induction of resistance through its effects on jasmonate-mediated signalling [12] but subsequent work indicated that the mechanism might be more complex [13].

Studies with viruses of the genus *Potyvirus* have also demonstrated that viruses can engender enhanced aphid performance on infected plants. This was seen in a variety of host-potyvirus combinations, including: turnip mosaic virus (TuMV) in *Arabidopsis thaliana* [14]; potato virus Y (PVY) in potato (*Solanum tuberosum*) [15], and zucchini yellow mosaic virus in zucchini squash (other names: marrow or courgette) (*Cucurbita pepo*) [16, 17]. Potential mechanisms proposed to explain enhanced aphid performance include improvement in the nutritional properties of the infected host, combined with inhibition of its defenses [14, 16, 17]. Casteel and colleagues [14] showed that, in Arabidopsis plants infected with TuMV, the viral NIa protein induced these changes in the host. However, a virus can induce opposite effects on host-vector interactions in different hosts. For example, rather than enhancing survival and reproduction, CMV induces feeding deterrence against *M. persicae* in Arabidopsis [18] and against *M. persicae* as well as *Aphis gossypii* in the ‘Dixie’ variety of squash and in cucumber (*Cucumis sativus*) [19, 20]. Similar contrasts have been noted for potyviruses. Hence, whereas PVY infection enhanced *M. persicae* feeding in potato [15] this virus induced resistance to this aphid in *N. benthamiana* [13]. Interestingly, PVY infection inhibited feeding on potato by another aphid, *Macrosiphum euphorbiae* [15]. The induction of resistance to *M. persicae* by CMV in Arabidopsis and by PVY in *N. benthamiana* appears to emerge from the combined action of multiple viral gene products [13, 18].

Mauck and colleagues [19] noted that despite being less palatable to aphids, squash plants infected with the Fny strain of CMV were initially more attractive to aphids and this was related to increases in the quantity of volatile organic compounds (VOCs) released by infected plants. They proposed that increased VOC emission acts as a deceptive semiochemical signal to lure aphids towards infected plants, while the unpalatability of the infected plants would repel the insects as soon as they had acquired virions during the initial probing of the host’s epidermal tissue. The effects of plant VOC emission on insect herbivore behavior can be profound but it is thought that changes in the composition of the VOC blend, rather than the quantity of VOCs emitted, is typically most important in determining changes in insect behavioral responses [21–23]. However, Mauck and colleagues [19] found that the VOC blend emitted by CMV-infected squash appeared to be qualitatively similar to the blend emitted by healthy plants.

Since VOC emission had such a profound influence on the interactions of aphids and squash plants infected with the Fny strain of CMV [19], we suspected that the same virus strain would also cause changes in the quantity or blend composition of VOCs emitted by tobacco plants. We hypothesized that increases or alterations in VOC blends might act as a non-deceptive signal that would attract aphids to tobacco plants that had been made more hospitable to colonization by virus infection. However, we found that although infection by either CMV or its mutant CMV∆2b did alter VOC emission in tobacco, these changes did not appear to increase the preference of aphids for infected versus mock-inoculated plants in free choice assays.

## Methods

### Biological materials

Tobacco (*Nicotiana tabacum* L.) cv. Xanthi seeds were sown and plants grown on Levington M3 compost (Scotts, Chillworth, Ipswich, UK). Seedlings were mechanically inoculated at the 3-to-4 leaf stage with purified virions of either CMV (strain Fny: [24]), or Fny-CMVΔ2b [25] diluted in water, or they were mock-inoculated with water. Inoculation efficiency was increased using Carborundum as an abrasive (SiC powder: Alfa Aesar, Heysham, U.K.). Plants were used for experiment at 10 days post-inoculation. Infection with CMVΔ2b in tobacco does not induce visible symptoms [26, 27], so infection was confirmed by double antibody sandwich enzyme-linked immunosorbent assay at the end of each experiment (Bioreba, AG, Reinach, Switzerland) or using CMV-specific Immunostrips (Agdia Inc., Elkhart, IN, USA) as described previously [10]. Experiments with aphids used wingless (apterous) *Myzus persicae* (Sulz.) of clone US1L [28] and *M. persicae* colonies were maintained on tobacco plants.

### Volatile organic compound collection and analysis

VOC collection by entrainment was done by placing plants singly in sealed 1.0 l glass chambers with charcoal-filtered air pumped in at the bottom of the vessel. All parts were cleaned with acetone and baked in an oven at 150 °C for 2 h before use. VOCs were captured on a Porapak Q filter [50 mg, 60/80 mesh size, Supelco (Sigma-Aldrich)] contained in a glass gas chromatograph (GC) inlet liner between silanized glass-wool plugs [29]. The Porapak Q tube was inserted at the top of the chamber, and headspace air was drawn through the tube at a rate of 750 ml.min^−1^ for a 3-day period. The entrained VOCs were eluted from the Porapak Q filters with 750 μl redistilled diethyl ether and stored at -20 °C. Quantitative VOC analysis was performed using a Hewlett-Packard (HP) 6890 GC equipped with a cold on-column injector, a flame ionisation detector and a 50 m × 0.32 mm internal diameter (I.D.) HP-1 bonded phase fused silica capillary column. The oven temperature was maintained at 30 °C for 2 min and then programmed at 5 °C.min^−1^ to 150 °C, followed by 10 °C.min^−1^ to 250 °C. The carrier gas was hydrogen [30, 31].

Compounds were identified by coupled GC-mass spectrometry (GC-MS) and comparison of retention times with authentic standards. A capillary GC column (50 m × 0.32 mm I.D. HP-1) fitted with an on-column injector was directly coupled to a mass spectrometer (Agilent 5973 MSD). Ionisation was by electron impact at 70 eV at 250 °C. The oven temperature was maintained at 30 °C for 5 min and then programmed at 5 °C.min^−1^ to 250 °C [30, 31]. The carrier gas was helium. Compounds were identified by comparison of the obtained spectra with a mass spectral database (National Institute of Standards and Technology mass spectral library version 2.0. Office of the Standard Reference Data Base, National Institute of Standards and Technology, Gaithersburg, Maryland: http://www.nist.gov). The isomeric composition of compounds was not investigated.

### Aphid free choice assays

Aphid free choice assays were performed using wingless adult *M. persicae* on tobacco plants infected systemically with CMV, CMVΔ2b, or plants mock-inoculated with water as controls. Plants were grown in square 33 × 33 cm plastic plant pots containing two plants planted equidistantly from the aphid placement point. Ten days following viral inoculation, 10–15 adult aphids were placed inside 1.5 ml microcentrifuge tube placed in the middle of the pot, equidistant between plants placed 9 cm apart. The lid of the tube was left open, allowing the aphids to escape. The numbers of aphids that settled on either plant were recorded after 15 min, 1 h and 24 h. To confine the aphids, each pot containing a pair of plants was wrapped in a micro-perforated bread bag (Associated Packaging Ltd., Tonbridge, Kent, UK) and was kept in a mesh insect cage (Insect Cage Net, Carmarthen, Dyfed, UK). Experiments were carried out under normal illumination six times with a total of *n* = 44–59 pairs of plants used for each comparison, and to exclude visual cues, experiments were carried out in the dark seven times with a total of *n* = 72–84 pairs of plants for each comparison. Plants grown and experimented upon under ‘normal illumination’ were maintained in a controlled environment room (Conviron Ltd., Winnipeg, Manitoba, Canada) with a 16 h photoperiod (200 μE.m^2^.s^−1^ of photosynthetically active radiation) at 22 °C and 60% relative humidity. For experiments carried out in darkness the same conditions of temperature and humidity were used but without light.

### Statistical analysis

To assess whether or not aphids preferentially settled on infected plants, we modelled the data from individual pots in a single choice test as independent samples from binomial distribution with a probability of “success” (i.e. choosing an infected plant) that was fixed for each experiment at each time. We chose to fix the probability of success for each experiment – which corresponds to pooling the data over all pots – after exploratory analysis, which indicated no over-dispersion in the data and so no need for a more complex (e.g. beta-binomial) model to account for systematic differences between pots. We therefore fitted the model *n*_*i*,*t*_^+^ ~ Bin(*n*_*i*,*t*_^+^ + *n*_*i*,*t*_^−^, *p*_*t*_) to the data, where *n*_*i*,*t*_^+^ is the number of aphids that settled on the infected plant in the *i* ^th^ pot at time *t*, and *n*_*i*,*t*_^−^ is the number that settled on the uninfected plant. For *t* = 0.25 h, *t* = 1 h and *t* = 24 h, the probability that aphids choose to settle on infected plants, *p*_*t*_ *,* is the parameter that must be estimated: there are therefore three probabilities to be estimated for each experiment (Eq. 1).

We calculated posterior distributions of *p*_0.25_, *p*_1_ and *p*_24_ in the Bayesian framework, using uninformative priors to allow estimates to be entirely driven by the data. The probability density of *p*_*t*_ given the experimental data, *D*, was therefore directly proportional to the likelihood function (i.e. the probability of the data given *p*_*t*_), with (for all three values of *t*)

1 $$ \mathrm{\mathbb{P}}\left({p}_t\Big| D\right)\propto \mathrm{\mathbb{P}}\left( D\Big|{p}_t\right)\propto {{\displaystyle {\prod}_i{p}_t}}^{n_{i, t}^{+}}{\left(1-{p}_t\right)}^{n_{i, t}^{-}}. $$

The level of support from the data for any preference in aphid choices was then assessed by checking whether or not 95% credible intervals for *p*_0.25_, *p*_1_ and *p*_24_ overlapped 0.5.

## Results

### Cucumber mosaic virus and its 2b protein affect volatile emission by tobacco

**Fig. 1:**
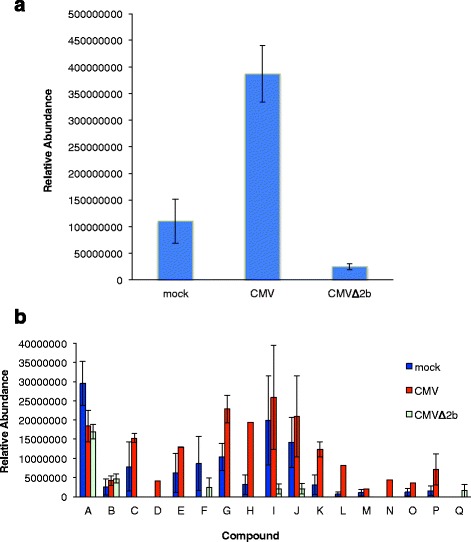
CMV infection quantitatively and qualitatively alters the blend of organic volatile compounds emitted by tobacco plants. Volatile organic compounds (VOCs) were collected by dynamic headspace trapping from tobacco plants that had been mock inoculated (mock) or inoculated with CMV (strain Fny) or its *2b* gene deletion mutant CMVΔ2b. **a** Total VOC emissions corrected for leaf area are shown as mean ± SEM. **b** Individual VOC emission corrected for leaf area are shown. A, 3-hexanol; B, *trans*-2-methylcyclopentanol; C, nonane; D, 2,5-dimethyl-3,4-hexanediol; E, 2,6-dimethyloctane; F, 6-methyl-5-hepten-2-one; G, limonene; H, non-2-en-1-ol; I, nonanal; J, decanal; K, undecanal; L, nerolidol; M, tetradecane; N, dodecanal; O, geranylacetone; P, α-farnesene, and Q, unknown [(potentially sulfur containing compound(s)]. VOCs were collected for 3 days (n = 3 plants per treatment group)

Headspace VOCs were collected from tobacco plants that had been mock-inoculated or infected with either CMV (strain Fny) or its *2b* gene deletion mutant (CMVΔ2b) and analysed by GC-MS. Quantitatively, VOC emission per unit of leaf area was approximately four-fold higher for CMV-infected plants than for mock-inoculated plants (Fig. 1a). The composition of the VOC blend emitted by CMV-infected plants differed qualitatively and quantitatively compared with mock-inoculated plants, with 15 compounds identified in the blend emitted by CMV-infected plants compared to 14 identifiable in the blend from mock-inoculated plants (Fig. 1b). Two compounds induced by CMV infection were absent from the VOC blend emitted by mock-inoculated plants (2,5-dimethyl-3,4-hexanediol and dodecanal) and the emission of at least one compound (6-methyl-5-hepten-2-one) was suppressed entirely in CMV-infected plants (Fig. 1b).

In tobacco CMVΔ2b accumulates to a lower titer than wild-type CMV and induces no discernible symptoms during a systemic infection (for examples, see refs. [26, 27]). Despite this, CMVΔ2b induced a striking decrease in the complexity of the VOC blend and in the overall quantity of VOCs emitted (Fig. 1a, b). The VOC profile of CMVΔ2b-infected plants was markedly impoverished compared to those of mock-inoculated and CMV-infected plants, indicating that the mutant virus suppressed the emission of the majority of compounds present in VOC profiles from CMV-infected or mock-inoculated plants (Fig. 1b). The VOC profile of CMVΔ2b-infected plants comprised only five identifiable compounds dominated by 3-hexanol and *trans*-2-methylcyclopentanol. There was evidence for emission of an unidentified VOC in the blend emitted by CMVΔ2b-infected plants, which was not present in the blends emitted by mock-inoculated or CMV-infected tobacco plants (Fig. 1b).

### Aphid free choice assay

**Fig. 2:**
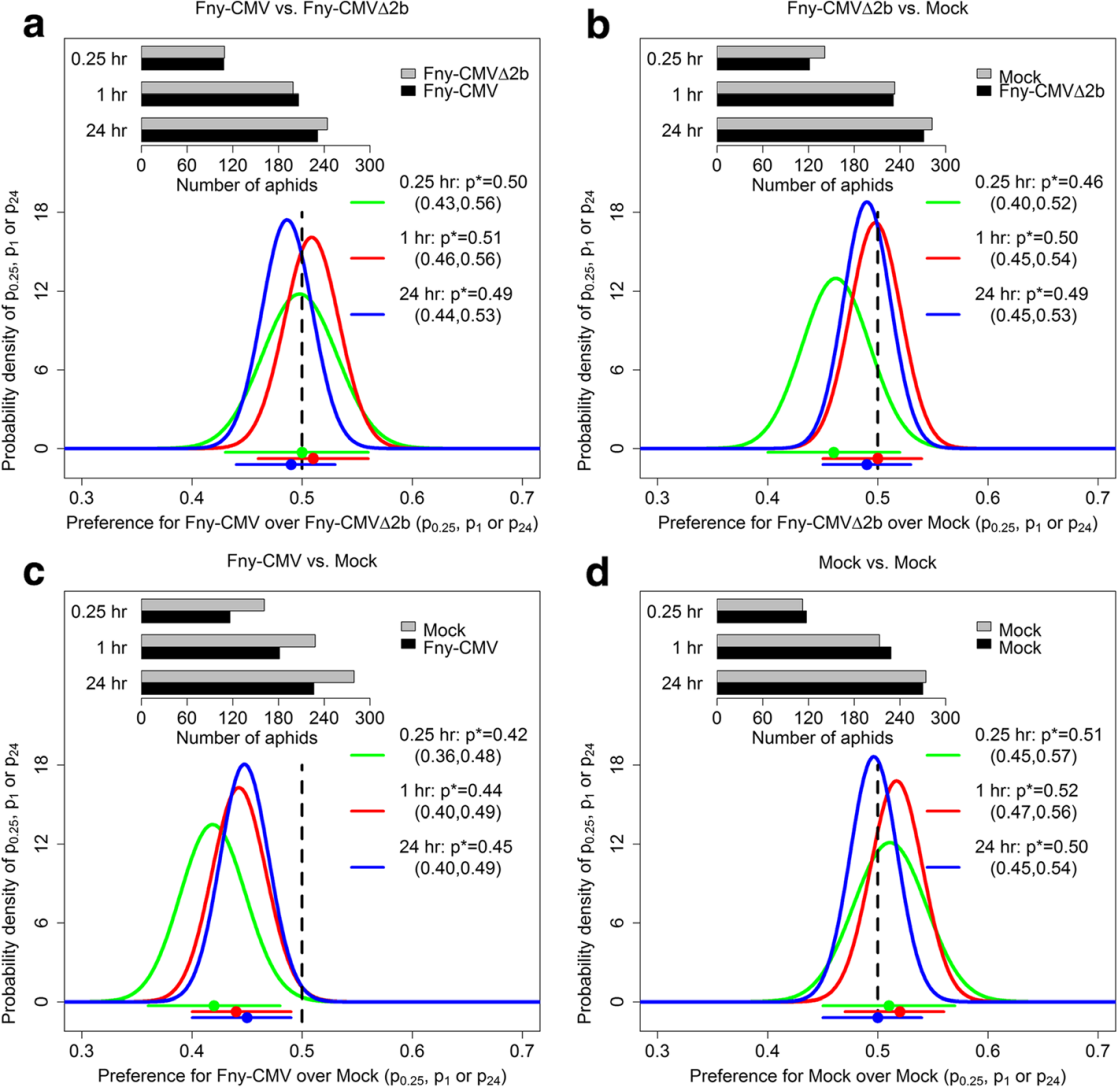
Settlement of aphids on infected tobacco under normal illumination. Pots contained two plants infected either with CMV strain Fny (Fny-CMV), the *2b* gene deletion mutant (Fny-CMVΔ2b), or a plant that had been mock inoculated with water (mock). Ten to 15 wingless adult aphids (*M. persicae*) were placed in a microfuge tube that was equidistant between each pair of plants (plants placed 9 cm apart). The number of aphids that settled on each plant was recorded at 0.25, 1 and 24 h after aphid release. Panels **a**-**d** show the combined data for six experiments carried out under normal illumination (16 h photoperiod). In each panel (**a**-**d**), the bar chart shows the experimental data; the lower graph shows the posterior density of the parameters *p*_0.25_, *p*_1_ and *p*_24_, the probabilities of choosing to settle on an infected plant conditional on settling at *t* = 0.25 h, *t* = 1 h and *t* = 24 h, respectively. The legend gives mean values and 95% credible intervals for each parameter: these are also shown graphically below the x-axis (dot is the mean; line shows the credible interval). Panel **c** shows that the data support a slightly reduced probability of aphids choosing to settle on CMV-infected plants over mock-inoculated ones at 0.25, 1 and 24 h (95% credible interval for the probability of settling on an infected plant conditional on settling was 0.36–0.48 for *p*_*0.25*_, 0.40–0.49 for *p*_1_, 0.40–0.49 for *p*_24_)

**Fig. 3:**
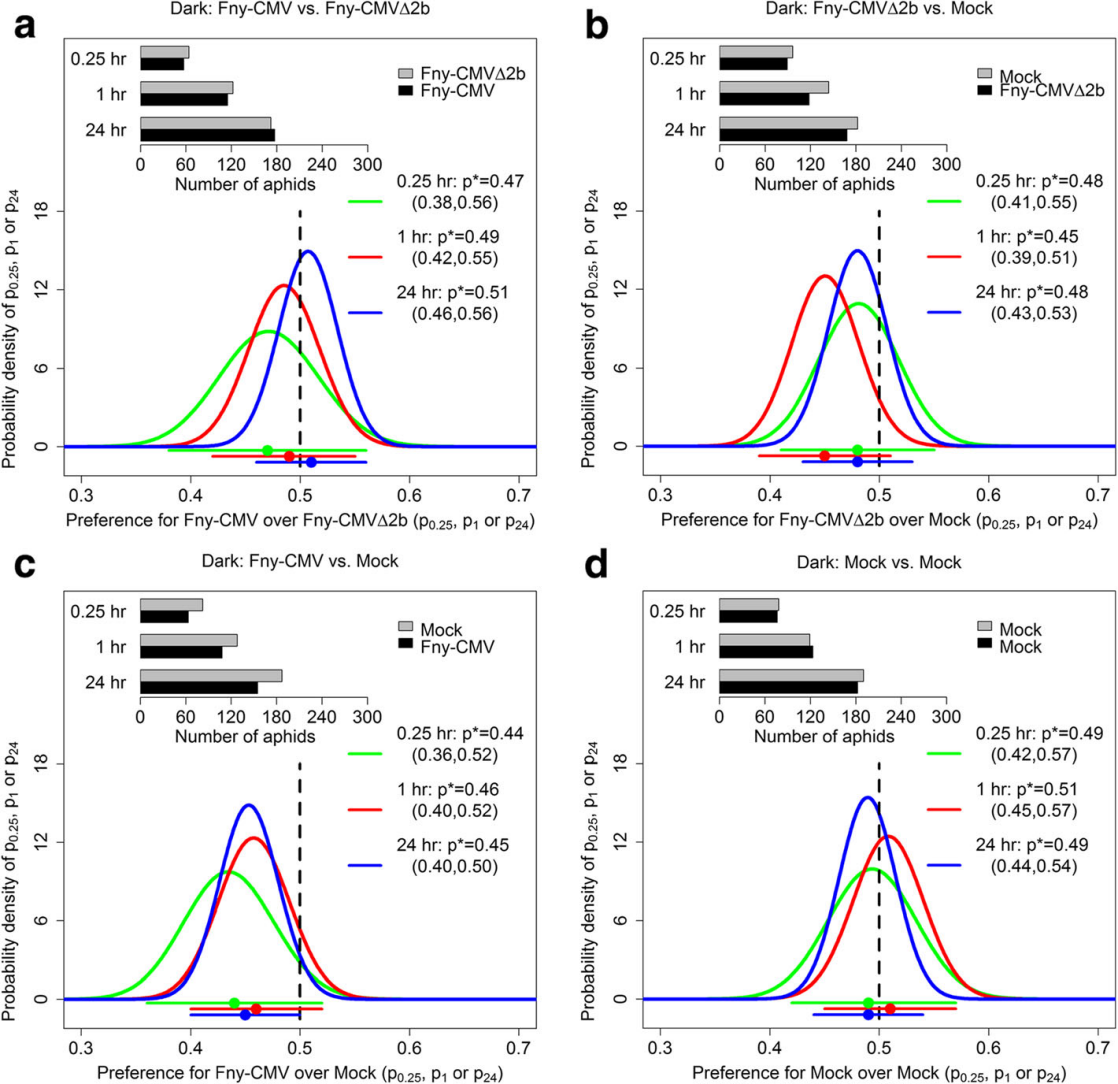
Aphids showed no preferential settling on CMV, CMVΔ2b or mock-inoculated tobacco plants in darkness. Pots contained two plants infected either with CMV strain Fny (Fny-CMV), the *2b* gene deletion mutant (Fny-CMVΔ2b), or a plant that had been mock inoculated with water (mock). Ten to 15 wingless adult aphids (*M. persicae*) were placed in a microfuge tube that was equidistant between each pair of plants (9 cm apart). The number of aphids that settled on each plant was recorded at 0.25, 1 and 24 h after aphid release. Panels **a**-**d**, show the combined data for seven experiments carried out in the dark. In each panel (**a**-**d**), the bar chart shows the experimental data; the lower graph shows the posterior density of the parameters *p*_0.25_, *p*_1_ and *p*_24_, the probabilities of choosing to settle on an infected plant conditional on settling at *t* = 0.25 h, *t* = 1 h and *t* = 24 h, respectively. The legend gives mean values and 95% credible intervals for each parameter: these are also shown graphically below the *x-axis* (dot is the mean; line shows the credible interval)

The free choice experiments with wingless aphids were carried out on six occasions under a normal illumination cycle (Fig. 2) and seven occasions in the dark to exclude visual cues (Fig. 3) (Additional file 1: Table S1) [32]. In the experiments carried out under normal illumination, aphids were found to be only marginally less likely to choose to settle on CMV-infected plants compared to mock-inoculated plants at all three time points (95% credible intervals for the probability of settling on an infected plant conditional on settling were 0.36–0.48 for *p*_*0.25*_, 0.40–0.49 for *p*_1_, 0.40–0.49 for *p*_24_) (Fig. 2c). However, there was no further decline in the number of aphids settling on CMV-infected plants from the 0.25 h time point through to the 1 h or 24 h assessment times (Fig. 2c). Thus, once they had settled on a plant, the aphids were not deterred from feeding.

**Fig. 4:**
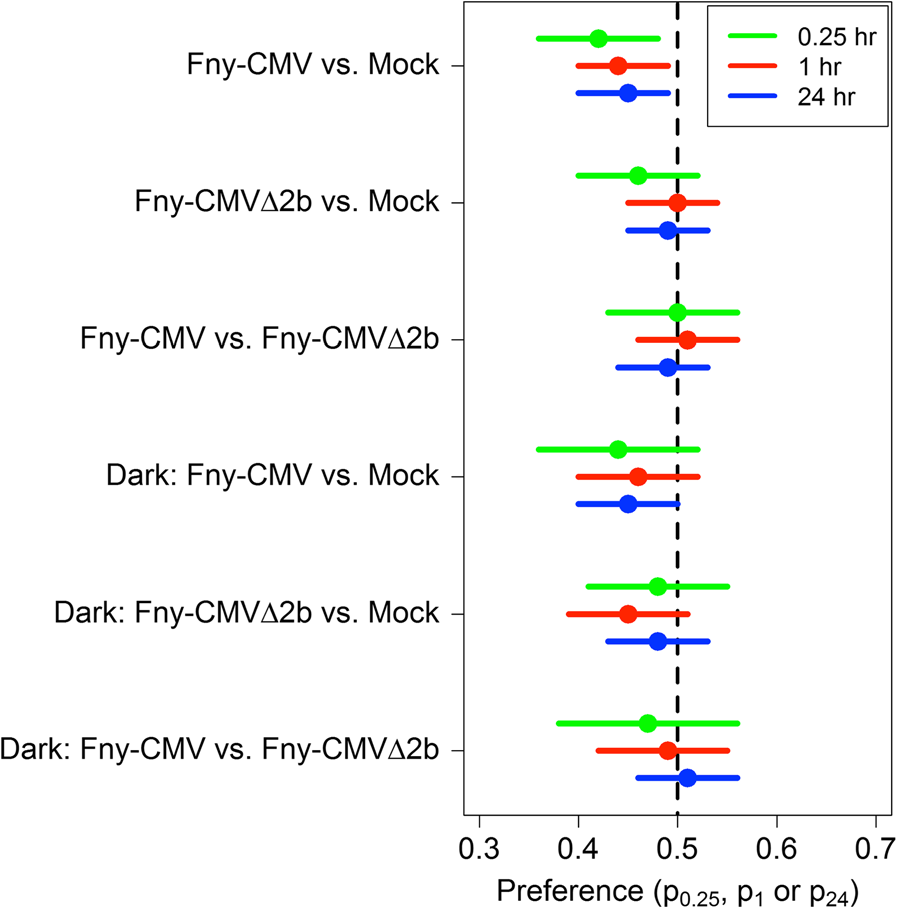
Summary of the aphid free choice assays performed under normal illumination and in darkness. Aphids (*M. persicae*) showed a marginally lesser tendency to settle upon plants infected with CMV than on mock-inoculated control plants at 0.25, 1 or 24 h post-placement when the experiment is done under normal illumination (95% credible interval for the probability of settling on an infected plant conditional on settling was 0.36–0.48 for *p*_*0.25*_, 0.40–0.49 for *p*_1_, 0.40–0.49 for *p*_24_) or under continuous darkness. Under continuous darkness aphids showed a tendency to discriminate against settling on plants infected with CMVΔ2b, although 0.5 was contained in all 95% credible intervals. Under normal illumination and in continuous darkness there was no apparent tendency for aphids to discriminate between CMV- or CMVΔ2b-infected plants. The labelling of the *y-axis* shows the pair of plants being tested (Fny-CMV, Fny-CMVΔ2b, and mock-inoculated plants) and the conditions (normal illumination cycle or continuous darkness) under which the test was carried out. The *x-axis* showed the range of the 95% credible interval of the parameters *p*_0.25_, *p*_1_ and *p*_24_, the probabilities of choosing to settle on an infected plant conditional on settling at *t* = 0.25 h, *t* = 1 h and *t* = 24 h, respectively

When the aphids were allowed to choose between settling on CMV-infected or on CMVΔ2b-infected plants, they showed no preference for one type of infected plant over the other (Fig. 2a). Aphids settled on mock-inoculated or CMVΔ2b-infected plants without showing any preferential settling (Fig. 2b) and they did not show any bias in choice tests with mock-inoculated plants only (Fig. 2d). Choice tests carried out in the dark (Fig. 3) indicated that under these conditions the aphids had no preferences for mock-inoculated plants, or for CMV-infected or CMVΔ2b-infected plants. For clarity, a graphical summary of the free choice experimental results performed under the normal illumination cycle (Fig. 2) and in darkness (Fig. 3) is presented in Fig. 4.

## Discussion

Previously, we showed that survival, reproduction, and duration of sustained ingestion from the phloem are all increased for *M. persicae* on tobacco (cv. Xanthi) infected with Fny-CMV [10]. We also showed that the *2b* gene deletion mutant Fny-CMVΔ2b induces antibiosis against aphids resulting in decreased aphid survival and reproduction [10]. In this study, we found Fny-CMV and Fny-CMVΔ2b infection induced distinct quantitative and qualitative changes to the VOC blends emitted by tobacco plants. Thus, in tobacco the 2b protein can influence the emission of VOCs, which has also been noted in tomato (*S. lycopersicum*) [33] and Arabidopsis [33, 34].

We had hypothesized that these changes in VOC output might influence aphid behaviour; for example, by making CMV or CMVΔ2b infected plants more or less attractive to aphids. If aphids made perfect choices to maximise their fitness, we would have expected them to be attracted by VOCs of CMV-infected plants and preferentially accumulate on diseased plants. This is because our previous studies showed that CMV infected plants are better quality hosts for aphids [10]. An alternative scenario is that CMV induces repellent volatiles to increase vector mobility and hence promote virus dissemination. However, free choice assays showed that aphids had at best only a marginally decreased probability of settling on CMV-infected versus mock-inoculated plants under a normal light/dark cycle and no detectable bias in continuous darkness. We conclude that it is unlikely that there is any biologically significant difference in *M. persicae* preference for virus-infected versus mock-inoculated tobacco plants in the dark or under normal illumination. This indicates that virus-induced changes in host VOC blends are not the only stimuli conditioning attractiveness of host plants and other cues such as virus-induced changes in optical characteristics [35] should be considered.

A fascinating possibility suggested by Mauck and colleagues [36] is that virus strains must co-evolve with hosts to exert complete ‘control’ over host-vector interactions. If this is correct, it may be that Fny-CMV is insufficiently adapted to tobacco to be able to manipulate this host’s VOC metabolism in a way that exerts biologically significant effects on vector behavior. However, for CMV there is little evidence for host-specific evolution of strains (most have very broad host ranges and wide geographic distributions) except in the case of one CMV strain that is highly adapted to rosemary (*Rosmarinus officinalis*). Curiously, this rosemary-adapted CMV strain is, unusually, very poorly transmissible by aphids [37]. An alternative hypothesis to explain our results with tobacco is that the virus-induced changes in VOC blends somehow decrease the attractiveness of CMV-infected plants. This might serve the interests of the virus since increased host quality might otherwise retard onward migration of viruliferous aphids.

CMVΔ2b infection makes tobacco plants poor hosts for *M. persicae* [10]. Despite this, aphids were not deterred from settling on CMVΔ2b-infected plants. Thus, virus-induced increases or decreases in host plant quality do not always correlate with changed attractiveness to vectors. It should also not be assumed that virus-induced changes in VOC emission affect all plant-insect interactions equally. For example, CMV-induced changes in VOC emission by squash plants attract aphids [19] but do not attract parasitoid wasps that prey on aphids [38], and infection of two Arabidopsis accessions with the LS strain of CMV did not alter aphid preferences in free choice assays [39].

These results support previous work [10] showing that CMV infection alters tobacco secondary metabolism and that the 2b protein has a role in this. However, changes in tobacco VOC emission do not act either as ‘honest’ advertisements to aphids for good hosts (in the case of CMV-infected plants) or ‘deceptive’ advertisements or warnings of poor hosts (as in the case of CMVΔ2b-infected plants). Presently, it is not known which specific CMV-induced biochemical changes affect host quality for aphids on tobacco. However, it is known that nicotine does not mediate CMVΔ2b-induced antibiosis [10]. In melon (*Cucumis melo*), CMV infection elevates phloem sugar levels [40], which might benefit aphids. However, if similar phloem sugar increases occur in *Cucurbita pepo* or *Cucumis sativus*, any beneficial effect on the aphids must be negated by other biochemical changes since CMV infection renders these plants unpalatable to *M. persicae* and *A. gossypii* [19, 20]. Another possibility is that amino acid levels increase in CMV-infected tobacco; this might explain enhanced aphid performance on TuMV-infected Arabidopsis [14]. Interestingly, in CMV-infected squash, which is an unsuitable host for aphids, phloem amino acid content is decreased [41].

CMV-infected cucurbits attract aphids through changes in VOC emission but the decreased palatability of these plants subsequently repels them [19, 20]. In Arabidopsis, CMV also induces unpalatability, which encourages aphid dispersion [18]. The effects of CMV on cucurbits and Arabidopsis probably drive spread of this non-persistently transmitted virus, since CMV acquisition and inoculation are favored by brief probe-feeds by aphids, not by prolonged ingestion [42]. In contrast, CMV infection of tobacco engenders protracted phloem feeding [10]. Contrasting effects of plant viruses in different host plants are also seen with PVY in potato (where *M. persicae* performance was enhanced) [15] and PVY in *N. benthamiana* (where resistance to *M. persicae* was induced) [13]. Indeed, one host can be affected in opposite ways by different non-persistently transmitted viruses. This is illustrated by Arabidopsis, which is more resistant or more susceptible to *M. persicae* when infected by, respectively, CMV [18] or TuMV [14]. Using the terminology of ‘Type 1’ (where viruses induce host resistance to aphid feeding) and ‘Type 2’, where viral manipulation inhibits resistance to aphids, we have suggested that Type 1 manipulation encourages transmission of non-persistently transmitted viruses, while Type 2 is a pay-back to the aphids that allows vector and virus reservoirs to persist within plant communities during times of stress [10, 18]. In this model, CMV-infected tobacco represents a Type 2 situation in which the virus inhibits host resistance to aphids.

## Conclusions

In tobacco, CMV and its 2b protein inhibit plant host resistance to aphids and alter the emission of VOCs by infected plants. Although infection of tobacco plants with CMV improves host quality for the aphids and alters VOC emission, it does not increase the attractiveness of infected plants to the insects. Thus, virus-bearing aphids will not be inhibited from migrating away from CMV-infected tobacco plants towards healthy plants.

## Additional file

Additional file 1: Table S1. Supplementary Data, Aphid Choice. (XLSX 83 kb)

## Abbreviations

CMV: Cucumber mosaic virus
CMV∆2b: 2b gene deletion mutant of CMV
GC-MS: Gas chromatography coupled mass spectrometry
PVY: Potato virus Y
TuMV: Turnip mosaic virus
VOC: Volatile organic compound
